# Supplementary material for: EnzML: multi-label prediction of enzyme classes using InterPro signatures
Source: BMC Bioinformatics. 2012 Apr 25;13:61. doi: 10.1186/1471-2105-13-61 (PMC3483700; doi:10.1186/1471-2105-13-61)
Supplement: Addtional file 5 — The Java code to format the data files, evaluate and predict. The file enzml_java_code.tar.gz contains the Java code used to format database data to ARFF and XML formats, to execute cross and train-test (jackknife) evaluations and to record evaluation results to database. More information is included in the readme.txt file and the Javadoc files. The code can be used with a MySQL database. To use a different database software, other JDBC drivers might be required. [file 1471-2105-13-61-S5.gz › java_code/utils/doc/uk/ac/ed/inf/utils/webutils/WebUtils.html]

WebUtils


---


|  |  |  |
| --- | --- | --- |
| **Overview** \| **Package** \| **Class** \| **Use** \| **Tree** \| **Deprecated** \| **Index** \| **Help** | |  |
| **PREV CLASS**   **NEXT CLASS** | **FRAMES**    **NO FRAMES**     **All Classes** |  |
| SUMMARY: NESTED \| FIELD \| CONSTR \| METHOD | DETAIL: FIELD \| CONSTR \| METHOD |  |


---


## uk.ac.ed.inf.utils.webutils Class WebUtils

```
java.lang.Object
  uk.ac.ed.inf.utils.webutils.WebUtils
```

---

``` public class WebUtils extends java.lang.Object ```

Web utilities. For reading from URL etc.

**Version:**
:   12 Dec 2007

**Author:**
:   Luna De Ferrari luna.deferrari-at-ed.ac.uk

---

| **Field Summary** | |
| --- | --- |
| `static boolean` | `m_debug` |


**Constructor Summary**

`WebUtils()`


| **Method Summary** | |
| --- | --- |
| `static java.io.BufferedReader` | `getReaderFromInputStream(java.io.InputStream stream)`             Creates a buffered reader from an input stream |
| `static java.io.InputStream` | `getStreamFromFileUri(java.lang.String url)` |
| `static java.util.zip.GZIPInputStream` | `getStreamFromGzipUrl(java.lang.String gzipUrl)` |
| `static java.io.InputStream` | `getStreamFromURL(java.lang.String url)`             Creates an input stream from a URL (gets all the HTML or XML contained in the page) |
| `static java.lang.String` | `getStringFromBufferedReader(java.io.BufferedReader reader)`             Get a string from a buffered reader |
| `static java.lang.String` | `getStringFromUrl(java.lang.String url, boolean gzip)`             Get a string from a URL |
| `static java.lang.String` | `md5Hash(java.lang.String s)`             Create the md5 hash of a text From http://snippets.dzone.com/posts/show/3686 |

**Methods inherited from class java.lang.Object**

`equals, getClass, hashCode, notify, notifyAll, toString, wait, wait, wait`

**Field Detail**

### m\_debug

```
public static boolean m_debug
```


**Constructor Detail**

### WebUtils

```
public WebUtils()
```


**Method Detail**

### getReaderFromInputStream

```
public static java.io.BufferedReader getReaderFromInputStream(java.io.InputStream stream)
```

:   Creates a buffered reader from an input stream

    :   **Parameters:**: `url` - a URL **Returns:**: reader **Throws:**: `java.io.IOException`

---


### getStreamFromFileUri

```
public static java.io.InputStream getStreamFromFileUri(java.lang.String url)
```

---


### getStreamFromGzipUrl

```
public static java.util.zip.GZIPInputStream getStreamFromGzipUrl(java.lang.String gzipUrl)
```

---


### getStreamFromURL

```
public static java.io.InputStream getStreamFromURL(java.lang.String url)
                                            throws java.io.IOException
```

:   Creates an input stream from a URL (gets all the HTML or XML contained in
    the page)

    :   **Parameters:**: `url` - a URL or file URI **Returns:**: an input stream object **Throws:**: `java.io.IOException`

---


### getStringFromBufferedReader

```
public static java.lang.String getStringFromBufferedReader(java.io.BufferedReader reader)
```

:   Get a string from a buffered reader

    :   **Parameters:**: `reader` - a reader object **Returns:**: **Throws:**: `java.io.IOException`

---


### getStringFromUrl

```
public static java.lang.String getStringFromUrl(java.lang.String url,
                                                boolean gzip)
```

:   Get a string from a URL

    :   **Parameters:**: `url` -: `gzip` - whether the document in the url has to be gunzipped **Returns:**

---


### md5Hash

```
public static java.lang.String md5Hash(java.lang.String s)
```

:   Create the md5 hash of a text From
    http://snippets.dzone.com/posts/show/3686

    :   **Parameters:**: `s` - the text to hash **Returns:**: the md5 code **Throws:**: `java.security.NoSuchAlgorithmException`


---


|  |  |  |
| --- | --- | --- |
| **Overview** \| **Package** \| **Class** \| **Use** \| **Tree** \| **Deprecated** \| **Index** \| **Help** | |  |
| **PREV CLASS**   **NEXT CLASS** | **FRAMES**    **NO FRAMES**     **All Classes** |  |
| SUMMARY: NESTED \| FIELD \| CONSTR \| METHOD | DETAIL: FIELD \| CONSTR \| METHOD |  |


---
